# Age-period-cohort analysis of lung cancer mortality inequalities in Southern Spain: missed opportunities for implementing equitable tobacco control policies

**DOI:** 10.1186/s12939-023-01946-y

**Published:** 2023-07-12

**Authors:** Juan Antonio Córdoba-Doña, Encarnación Benítez-Rodríguez, Antonio Escolar-Pujolar, Vanessa Santos-Sánchez

**Affiliations:** 1 Preventive Medicine and Public Health Unit, Hospital Universitario de Jerez, Jerez de la Frontera (Cádiz), Jerez de la Frontera, Spain; 2 Instituto de Investigación e Innovación Biomédica de Cádiz, INIBiCA, Cádiz, Spain; 3 Preventive Medicine and Public Health Unit, Hospital Universitario Puerta del Mar, Cádiz, Spain; 4 Departamento de Sociología, Trabajo Social y Salud Pública, Universidad de Huelva, Huelva, Spain

**Keywords:** Inequalities, Socioeconomic factors, Mortality, Lung cancer, Gender, Tobacco policies, Spain

## Abstract

**Background:**

Lung cancer mortality in European countries shows different epidemiological patterns according to sex and socioeconomic variables. Some countries show decreasing rates in both sexes, while others show a delayed profile, with increasing mortality in women, inconsistently influenced by socioeconomic status. Our aim was to evaluate the effect of age, period and birth cohort on lung cancer mortality inequalities in men and women in Andalusia, the southernmost region in Spain.

**Methods:**

We used the Longitudinal Database of the Andalusian Population, which collects demographic and mortality data from the 2001 census cohort of more than 7.35 million Andalusians, followed up between 2002 and 2016. Mortality rates were calculated for men and women by educational level, and small-area deprivation. Poisson models were used to assess trends in socioeconomic inequalities in men and women. Finally, age-period-cohort (APC) models were used separately for each educational level and gender.

**Results:**

There were 39,408 lung cancer deaths in men and 5,511 in women, yielding crude mortality rates of 78.1 and 11.4 × 10^5^ person-years, respectively. In men higher mortality was found in less educated groups and inequalities increased during the study period: i.e. the rate ratio for primary studies compared to university studies increased from 1.30 (CI95:1.18–1.44) to 1.57 (CI95:1.43–1.73). For women, educational inequalities in favour of the less educated tended to decrease moderately. In APC analysis, a decreasing period effect in men and an increasing one in women were observed. Cohort effect differed significantly by educational level. In men, the lower the educational level, the earlier the peak effect was reached, with a 25-year difference between the least-educated and college-educated. Conversely, college-educated women reached the peak effect with a 12-year earlier cohort than the least-educated women. The decline of mortality followed the same pattern both in men and women, with the best-educated groups experiencing declining rates with earlier birth cohorts.

**Conclusions:**

Our study reveals that APC analysis by education helps to uncover changes in trends occurring in different socioeconomic and gender groups, which, combined with data on smoking prevalence, provide important clues for action. Despite its limitations, this approach to the study of lung cancer inequalities allows for the assessment of gaps in historical and current tobacco policies and the identification of population groups that need to be prioritised for public health interventions.

## Background

Lung cancer is a very relevant disease worldwide, ranking first among causes of death from cancer (18% of total deaths) and the second in incidence (11.4% of total cancer cases) [1, 2]. In industrialised countries, there is an increase or stabilisation of the incidence in women and a decrease in men [3], although these trends are not uniform in all countries [4].

In Europe different epidemiological patterns according to gender have also been observed for many years. In general, there are divergent trends in mortality by sex, with incidence and mortality decreasing in men and increasing in women [5]. Indeed, most countries in Eastern, Western and Southern Europe still report increasing mortality in women [6]. More specifically, a ‘lagged’ pattern is observed in Southern Europe in relation to the north. In the northern countries, the incidence in women has reached its peak and is already on a downward trend, whereas in the south, it continues to increase [1]. Spain is the European country with the highest average annual growth in lung cancer mortality in women in the last two decades (4.1% per year). In men, the decrease has been 1.4% per year during this period [7].

Socioeconomic inequalities in lung cancer morbidity and mortality have also been known for decades. A negative association between socioeconomic status in men and a positive one in women have usually been reported [8]. These inequalities have been associated with structural factors such as social class, occupation and gender, which provide the social context for socialization and experiences that, in turn, influence individual choices to encourage or discourage tobacco use [9].

Although there are other factors beyond smoking in the etiopathogenesis of lung cancer, such as air pollution [10, 11] or occupational exposures to carcinogens [12], tobacco consumption is by far the leading cause of lung cancer.

The considerable weight of tobacco in the epidemic of lung cancer [13] suggests that the observed diversity of gender and socioeconomic patterns, with various lags, may be related to tobacco control policies, which are often directed at a general public, without a gender or social determinants of health approach being taken. For instance, there has been little recognition of the importance of understanding the context and challenges of women’s smoking and exposure to second hand smoke. Besides that, when population-based comprehensive interventions for tobacco control such as increasing prices of tobacco products, enforcing smoke-free laws, restricting promotion, conducting media campaigns and offering cessation treatments are not fully implemented to reach all population groups equally, they may exacerbate existing disparities [14, 15].

Outlining the magnitude of social inequalities in cancer and tracking progress in reducing them is recommended as a research priority [16]. Important trend changes in inequalities have also been revealed [17, 18]. We therefore contend that studies are needed that take into account birth cohort effects and the evolution of socioeconomic inequalities in lung cancer morbidity and mortality.

Our objective is to reveal trends in social inequalities by sex in different birth cohorts in a Southern European region with a ‘lagged’ epidemiological pattern and high tobacco-attributable mortality, [19], which may require a specific approach in public health interventions, and more specifically, in strategies to reduce tobacco use. Moreover, we are interested in revealing whether these inequalities are more related to contextual factors in the area of residence or to the characteristics of individuals, and in detecting population subgroups in which public health measures seem to be less effective.

## Methods

### Aim

Our aim was to evaluate the effect of age, period and birth cohort on lung cancer mortality inequalities in men and women aged 30 years and older in Andalusia, the southernmost region in Spain, from 2002 to 2016.

### Design

We used the Longitudinal Database of the Andalusian Population (LDAP), which started with the population registered in the 2001 Population and Housing Census (7,357,547 individuals) residing in Andalusia on the 1 January 2002 [20, 21]. This population was tracked until 31 December 2016. The LDAP merges information from the 2001 Population and Housing Census with events recorded in the Natural Population Movement (NPM) database, such as deaths, births, marriages and changes in the residential status that have occurred since 2002. The end of follow-up could be the result of (i) a death registered in the NPM, (ii) emigration outside Andalusia or (iii) censorship due to termination of the study.

### Setting

This study was carried out in Andalusia, the most populated region in Spain, which had 8,403,936 inhabitants in 2016. Economic indicators are largely below the European average, with the region having the highest poverty rate (32.3% in 2021) among the Spanish autonomous communities. Health indicators are also well under the average, with a high mortality rate in both men and women compared to the rest of the regions.

Lung cancer is the first cause of cancer mortality (19.5% of cancer-related deaths) and the fourth most frequent cancer in Spain [22]. Mortality rates in women continue to increase annually, while consistently trending downward in men [23]. In Andalusia, an east-west mortality pattern in men has been detected for several cancer types, including lung cancer, with higher mortality rates in the west. Moreover, an association between deprivation at the small area level and lung cancer mortality in men has been shown. This association is negative for women [24]. In 2017, lung cancer accounted for more than 30% of tobacco-attributable mortality in Andalusia among those 35 years and older [19].

In Spain and Andalusia, smoking was a widespread practice, with a smoking rate of more than 60% among men until 1980. This percentage has been decreasing since then, and in 2020, 26% of Spanish men considered themselves to be current smokers [25]. In women, tobacco consumption was very low until 1970, increased rapidly until 1990, and has been decreasing since then. In 2020, 19% of Spanish women were smokers [25].

### Population and variables

The initial census population of 7,357,547 individuals was tracked for 15 years until December 2016, yielding 98,842,980.9 person-years of follow-up (48,415,311.3 men-years and 50,427,669.6 women-years). Individuals were living in 5,381 census tracts corresponding to 770 municipalities in 8 provinces.

### Assessment of variables

The outcome of this study was individual lung cancer mortality as assessed from the time of the 2001 census until 31 December 2016. The locations of tumours analysed corresponded with the International Classification of Diseases (10th rev.) codes C33 and C34, malignant neoplasm of trachea, bronchus and lung.

We considered 3-year age groups (i.e. 12–14, 15–17, 18–20, 21–23, and so on) and time intervals for calculating death rates were divided into five three-year periods (2002–2004, 2005–2007, 2008–2010, 2011–2013 and 2014–2106). We classified educational level into five categories: very low (illiterate or less than one year of formal education), primary (elementary school, i.e. 6 to 8 years of formal education), secondary first circle (elementary baccalaureate or similar degree), secondary second circle (up to 12 years of formal education) and university studies. Information was available at the census tract level. Therefore, data were structured in groups defined by two sex categories (men and women), 34 age categories, 5 education categories, 5 time periods and 5,381 census tracts, yielding 9,147,700 groups. Each group had its corresponding number of person-years and count of lung cancer deaths.

To assess the contextual socioeconomic status, we used a deprivation index (DI) at the census tract level, the same for both men and women [26]. The index was constructed with data from the 2001 Population and Housing Census regarding (i) percentage of people with low educational level, (ii) percentage of unskilled workers and (iii) unemployment rate. We carried out a principal component analysis to calculate the DI that separated the census tracts into five levels of deprivation, according to the quintiles of the respective factorial scores. Census tracts with the lowest social deprivation were designated level 1, and those with the highest social deprivation were designated level 5.

### Statistical analyses

First, the number of lung cancer deaths and person-years of follow-up were presented for period, educational level and DI, separately for each sex. In a second step, world population age-adjusted rates [27] by period, educational level and deprivation quintile were calculated. Third, three-year group age-specific rates were estimated for each time period, and lung cancer mortality rates by age group and birth cohort were estimated for both men and women. In a fourth step, we used Poisson regression models to assess temporal trends in inequalities, using education and deprivation as socioeconomic variables.

Finally, we used an age-period-cohort analysis trying to uncover the diverse birth-cohort and period effects related to the epidemiology of lung cancer in different socioeconomic groups. Age-period-cohort modelling is a well-known quantitative method used to improve understanding of disease trends by attempting to unravel the factors influencing all ages. In addition to the evaluation of age effects as more related to biological or social factors, these models allow researchers to assess period effects, such as changes in medical practice or in public health policies that occur simultaneously, and cohort effects, which are related to circumstances that affect an entire generation, such as similar behaviours, exposures to risk or protective factors [28]. In this case, in order to extend our research with a focus on social inequalities in health, we carried out an age-period-cohort analysis according to educational level.

These models suffer from an identifiability problem, since cohort = period-age. The literature reports different approaches to solve this drawback. We used Rutherford’s Stata package “apcfit“[29], which is based on Carstensen’s method [30], and which takes age, period and cohort as continuous variables using appropriate cubic spline functions in the framework of a generalised linear model. We used diverse parametrizations for the models, and chose the best-fitting following the Akaike information criterion. In our parametrization, age effects were expressed as rates, period effects as rate ratios relative to the reference period, and cohort effects as rate ratios constrained to be 0 on average on the log scale.

All analyses were performed using Stata software version 16.

## Results

There were 39,408 lung cancer deaths in men and 5,511 in women over 48,415,311.3 men-years and 50,427,669.6 women-years between January 2002 and December 2016, yielding a crude mortality rate of 78.1 × 10^5^ person-years in men and 11.4 × 10^5^ person-years in women (Table 1). World population age adjusted rates by period, educational level and deprivation quintile are also reported in Table 1. Between 2002 and 2004 and 2014–2016, we observed an increasing trend in mortality rates in women, from 4.3 × 10^5^ to 6.9 × 10^5^, and a clear downward trend in mortality rates in men, which declined from 53.9 × 10^5^ to 42.6 deaths x 10^5^ person-years. With regard to educational level, we found higher rates in more educated groups of women (secondary second cycle and university studies − 10.4 and 9.1 × 10^5^ respectively) than in the least-educated women (5.1 × 10^5^). On the contrary, in men, we detected a quasi-linear negative association between education and lung cancer mortality rate, with highest rates in the less-educated groups (59.4 × 10^5^ in men with no education versus 35.2 × 10^5^ in college-educated men). A similar pattern was observed in the distribution of mortality rates across deprivation quintiles: among women, higher rates were observed in the less deprived group, while in men mortality is directly associated with deprivation. Absolute differences observed among education levels were wider than those observed among deprivation strata, both in men and women.

**Table 1:** Age adjusted mortality rates in men and women by period, education and deprivation. Andalusia, 2002–2016

			Women			Men	
		Deaths	Person-years	Adjusted rate x 10^5^ p-y (CI95)	Deaths	Person-years	Adjusted rate x 10^5^ p-y (CI95)
Total		5511	48415311.3	5.85 (5.69–6.02)	39,408	50427669.6	48.43 (47.94–48.91)
Period	2002–2004	768	10750960.2	4.35 (4.02–4.68)	7911	10413859.4	53.86 (52.65–55.08)
	2005–2007	915	10419750.5	4.99 (4.64–5.33)	7894	10044367.9	51.06 (49.91–52.21)
	2008–2010	1088	10096467.9	5.91 (5.53–6.28)	7869	9690363.0	48.23 (47.15–49.32)
	2011–2013	1317	9766758.5	6.76 (6.37–7.15)	8062	9332666.1	47.27 (46.21–48.32)
	2014–2016	1423	9393732.5	6.92 (6.54–7.31)	7672	8934054.9	42.65 (41.68–43.63)
Educational level	University	522	4913081.1	9.06 (8.22–9.90)	2475	4499818.6	35.17 (33.76–36.58)
	Secondary 2nd cycle	509	5658755.5	10.44 (9.48–11.40)	2510	5983027.0	41.05 (39.39–42.70)
	Secondary 1st cycle	1184	12746858.2	6.89 (6.49–7.30)	6641	13055912.4	43.64 (42.54–44.74)
	Primary	1300	12231767.2	5.14 (4.84–5.44)	10,308	12134376.8	49.68 (48.71–50.66)
	No studies	1996	14877208.9	5.07 (4.64–5.50)	17,474	12742177.5	59.40 (58.21–60.59)
Deprivation	Lowest deprivation	1604	11401609.8	7.90 (7.51–8.30)	6603	10562588.8	39.78 (38.82–40.74)
	Low	1224	10903485.6	6.24 (5.88–6.61)	7589	10315334.1	46.27 (45.22–47.33)
	Intermediate	1029	10305849.7	5.26 (4.92–5.61)	8425	9908648.7	51.18 (50.06–52.29)
	High	920	9438867.1	5.00 (4.65–5.36)	8559	9233083.2	52.70 (51.54–53.85)
	Highest deprivation	734	8377857.3	4.21 (3.87–4.55)	8232	8395656.3	53.78 (52.56-55.00)

**Fig. 1:**
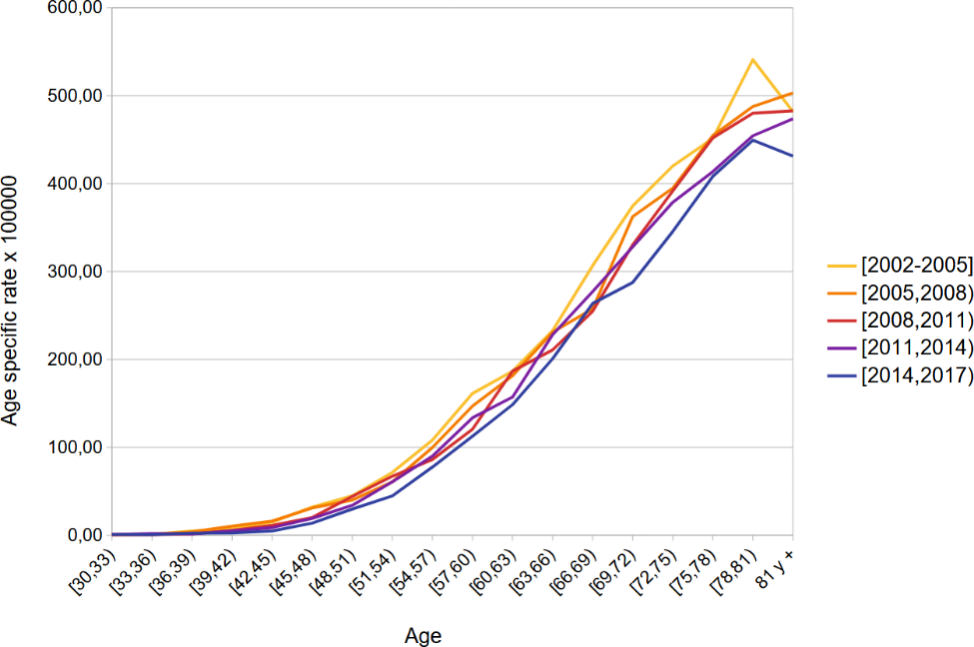
Lung cancer mortality age specific rates by period. Andalusian men 30 and older. 2002–2016

**Fig. 2:**
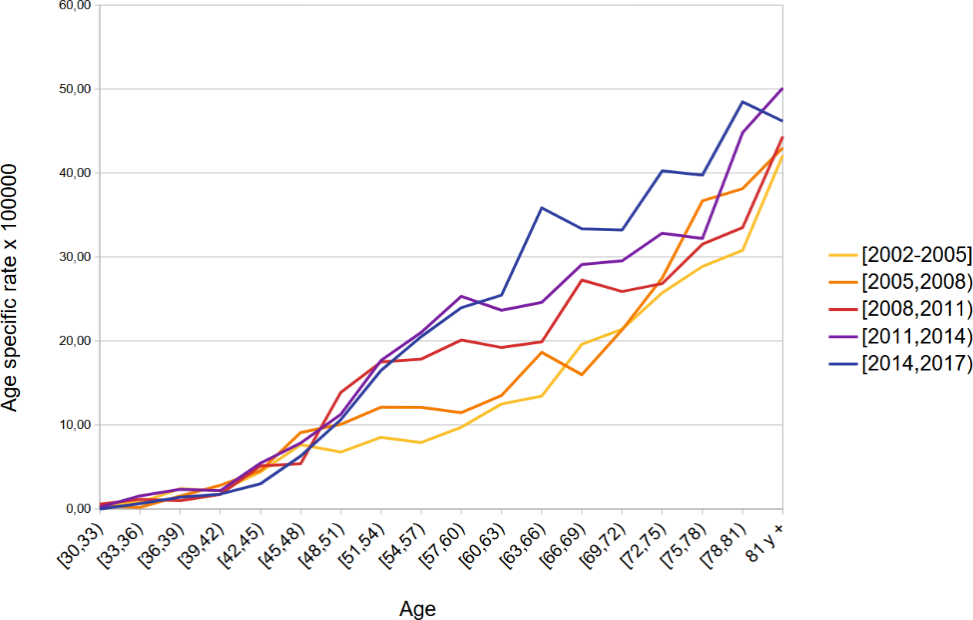
Lung cancer mortality age specific rates by period. Andalusian women 30 and older. 2002–2016

Figures 1 and 2 show age specific lung cancer death rates estimated for each time period for men and women. Mortality rates rose dramatically with age in both sexes. As expected, a decreasing time trend was observed in most 3-year age groups in men, especially in those over 70 years of age. On the other hand, in women, there was an increasing trend in age-specific mortality rates in those over 60 years of age throughout the entire period, while the rates remained stable or decreased slightly in women between 50 and 60 years of age in the last period (2014–2016) compared to the previous one.

**Fig. 3:**
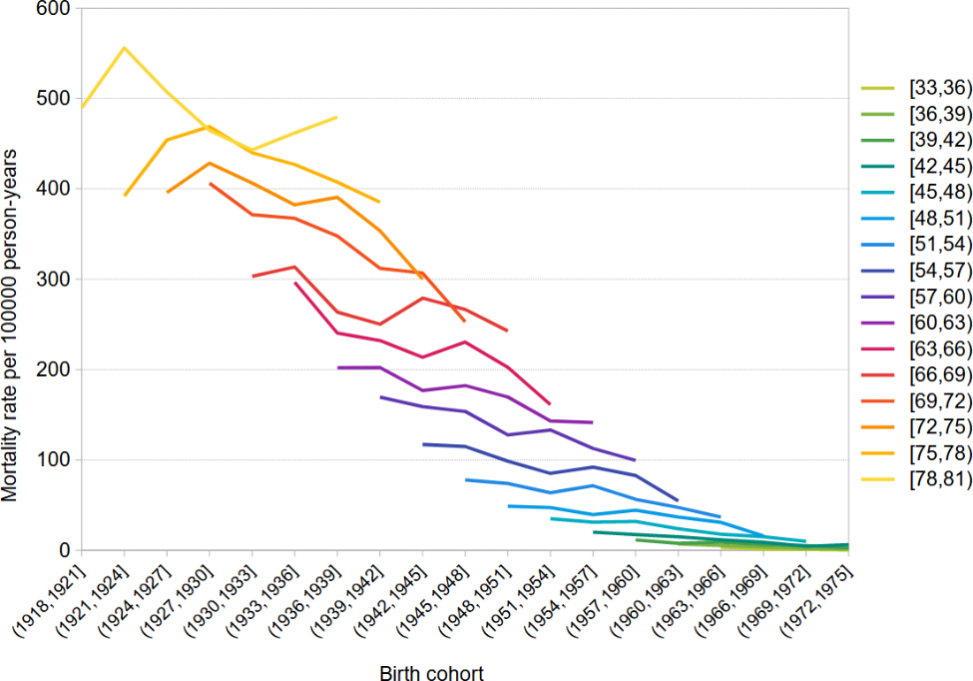
Cohort specific lung cancer mortality rates by age. Andalusian men, 2002–2016

**Fig. 4:**
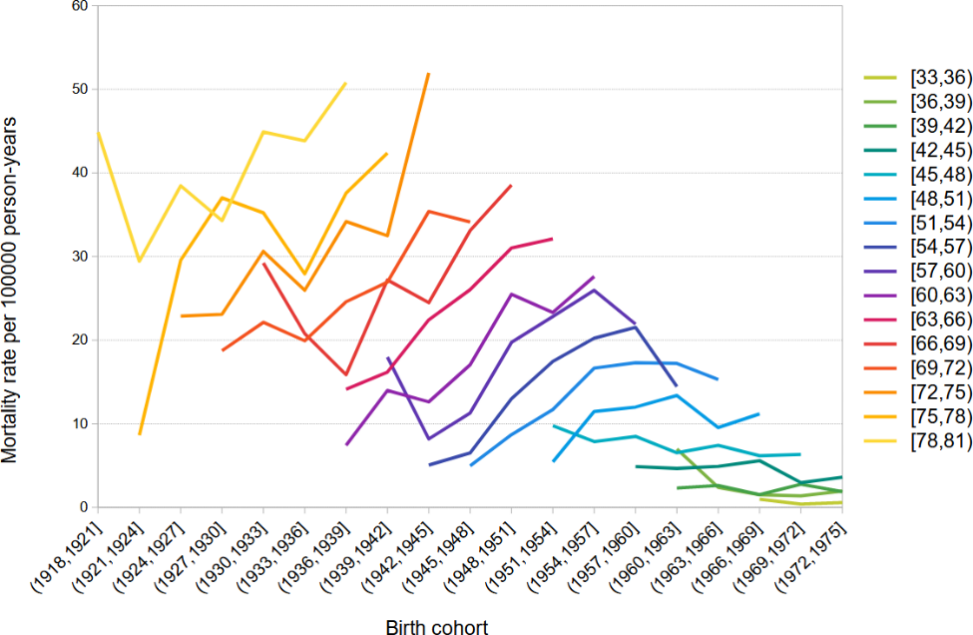
Cohort specific lung cancer mortality rate by age. Andalusian women, 2002–2016

Birth cohort lung cancer mortality rates by age group for both men and women are shown in Figs. 3 and 4. In Andalusian men, we observed declining lung cancer mortality rates over birth cohorts in all age groups. In contrast, in women, we found a clear upward trend in rates over birth cohorts in women aged 48 and older, but a declining trend over birth cohorts among women aged 30 to 47. There was lower mortality in recent birth cohorts in this younger age interval.

We used age adjusted Poisson regression models to assess temporal trends in inequalities, separately using education and deprivation as socioeconomic variables and using person-years as offset, both for men and women. Mortality rate ratios for each period were estimated by taking the university studies group and the least deprived groups as reference levels, respectively. Results are reported in Table 2. Among men, relative negative educational differences in mortality increased between the first and last period. For instance, the mortality rate ratio for men who had undertaken primary studies as compared to university studies increased from 1.30 (CI95: 1.18–1.44) to 1.57 (CI95: 1.43–1.73) between 2002–2004 and 2014–2016, and, for men with no studies, it changed from 1.52 (CI95: 1.38–1.69) to 1.76 (CI95: 1.60–1.94) in the same time span, thus indicating increasing educational inequalities in men. For women, on the contrary, educational inequalities were positive, and tended to decrease very moderately, and only among intermediate educational groups and the reference group. For instance, the mortality rate ratio for women with secondary-first cycle studies decreased from 0.67 (CI95: 0.48–0.93) to 0.87 (CI95: 0.72–1.05) during the study period. Compared to inequalities observed using educational attainment as a socioeconomic status variable, relative inequalities in mortality using deprivation as socioeconomic status variable were less pronounced in men (IRR for most deprived compared to least deprived = 1.36; CI95: 1.26–1.46) and negligible in women.

**Table 2:** Trends in educational and deprivation inequalities in lung cancer mortality by sex. Andalusia, 2002–2016

	Women					Men				
	AAMRR (CI95%)	AAMRR (CI95%)	AAMRR (CI95%)	AAMRR (CI95%)	AAMRR (CI95%)	AAMRR (CI95%)	AAMRR (CI95%)	AAMRR (CI95%)	AAMRR (CI95%)	AAMRR (CI95%)
**Education**	2002–2004	2005–2007	2008–2010	2011–2013	2014–2016	2002–2004	2005–2007	2008–2010	2011–2013	2014–2016
University	reference	reference	reference	reference	reference	reference	reference	reference	reference	reference
Sec 2nd cycle	1.52 (1.06–2.17)	1.11 (0.80–1.55)	1.18 (0.89–1.56)	1.11 (0.88–1.40)	1.07 (0.85–1.35)	1.08 (0.94–1.23)	1.12 (0.99–1.28)	1.20 (1.06–1.36)	1.16 (1.03–1.30)	1.31 (1.18–1.47)
Sec 1st cycle	0.67 (0.48–0.93)	0.77 (0.58–1.01)	0.79 (0.62–0.99)	0.78 (0.64–0.95)	0.87 (0.72–1.05)	1.19 (1.06–1.33)	1.26 (1.13–1.40)	1.29 (1.16–1.43)	1.23 (1.12–1.35)	1.33 (1.21–1.47)
Primary	0.56 (0.41–0.77)	0.58 (0.44–0.77)	0.63 (050 − 0.80)	0.56 (0.46–0.69)	0.65 (0.53–0.78)	1.30 (1.18–1.44)	1.35 (1.22–1.50)	1.54 (1.39–1.70)	1.41 (1.28–1.54)	1.57 (1.43–1.73)
No studies	0.60 (0.44–0.81)	0.59 (0.45–0.77)	0.58 (0.46–0.74)	0.48 (0.39–0.59)	0.56 (0.45–0.67)	1.52 (1.38–1.69)	1.56 (1.41–1.72)	1.70 (1.54–1.87)	1.56 (1.43–1.71)	1.76 (1.60–1.94)
**Deprivation**										
Lowest	reference	reference	reference	reference	reference	reference	reference	reference	reference	reference
Low	0.85 (0.70–1.04)	0.78 (0.65–0.94)	0.67 (0.57–0.80)	0.86 (0.74-1.00)	0.80 (0.71–0.94)	1.15 (1.07–1.24)	1.18 (1.10–1.27)	1.20 (1.11–1.29)	1.11 (1.03–1.19)	1.17 (1.08–1.25)
Intermediate	0.64 (0.52–0.80)	0.69 (0.57–0.84)	0.67 (0.56–0.80)	0.69 (0.58–0.81)	0.73 (0.63–0.85)	1.26 ( 1.17–1.35)	1.22 (1.13–1.31)	1.36 (1.26–1.46)	1.28 (1.19–1.37)	1.27 (1.18–1.37)
High	0.60 (0.48–0.75)	0.66 (0.55–0.81)	0.64 (0.53–0.76)	0.64 (0.54–0.76)	0.67 (0.57–0.788)	1.30 (1.21–1.39)	1.33 (1.23–1.42)	1.35 (1.26–1.45)	1.29 (1.20–1.38)	1.27 (1.19–1.37)
Highest	0.58 (0.46–0.73)	0.60 (0.49–0.75)	0.51 (0.42–0.62)	0.58 (0.48–0.69)	0.58 (0.48–0.69)	1.28 (1.19–1.38)	1.33 (1.23–1.43)	1.36 (1.26–1.46)	1.27 (1.18–1.37)	1.36 (1.26–1.46)

*AAMRR: Age adjusted mortality rate ratio

**Fig. 5:**
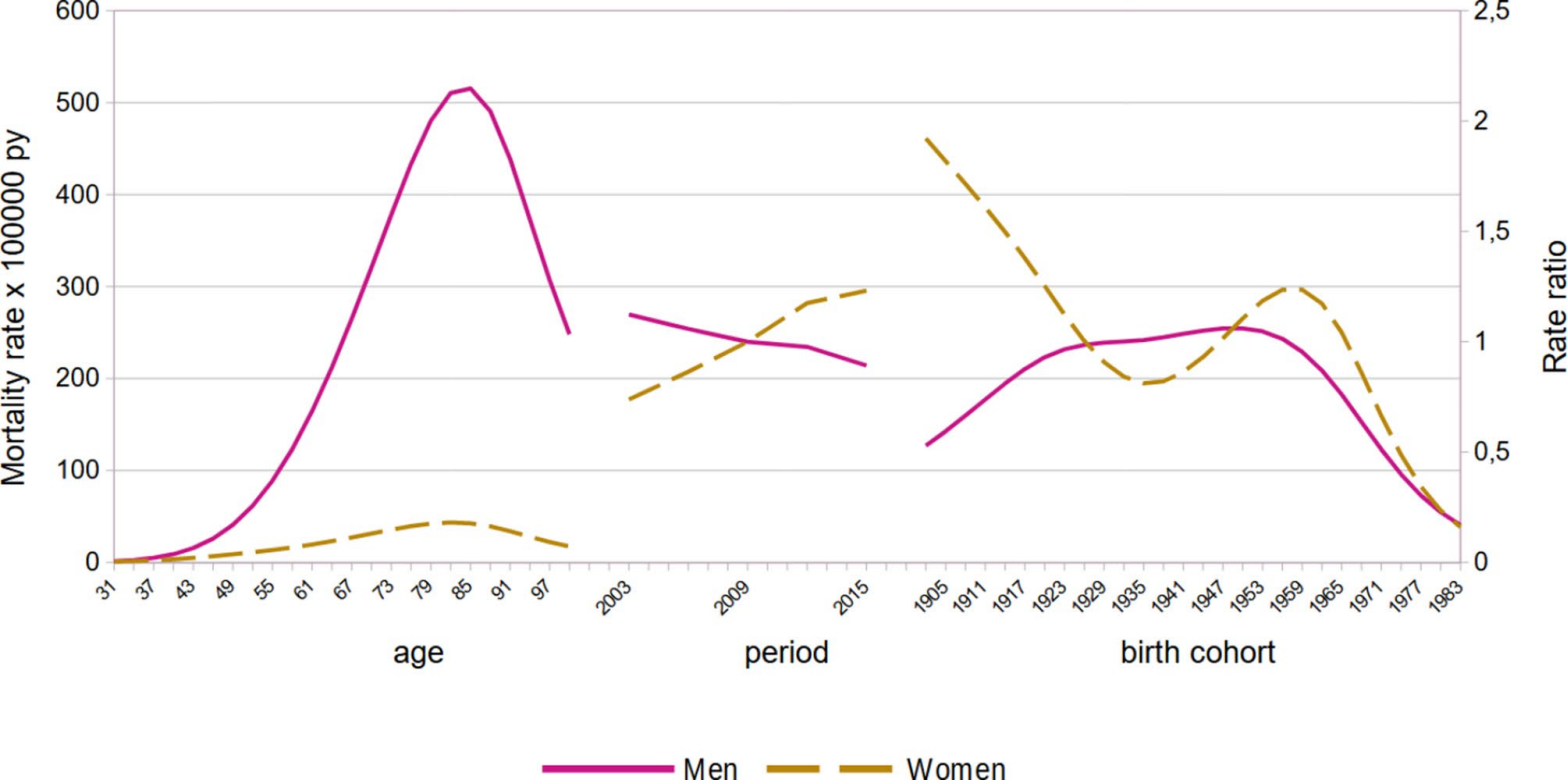
Age-Period-Cohort analysis for lung cancer mortality. Men and women. Andalusia, 2002–2016

The estimates of age period cohort effects on lung cancer mortality for men and women are shown in Fig. 5. Mortality increased with age, peaking at around 80 years of age in both sexes. It is worth noting we observed a decreasing period effect in men but a clearly increasing period effect in women. In men, there was an increase in the cohort effect from those born at the beginning of the 20th century to the cohort born in 1920. After this, the effect stabilised until a decline began with the cohort born in 1955. In women, rate ratios decreased until the cohort born in 1935. From that cohort onwards, the effect increased in subsequent cohorts before beginning to decline with the cohort of women born in 1960.

**Fig. 6:**
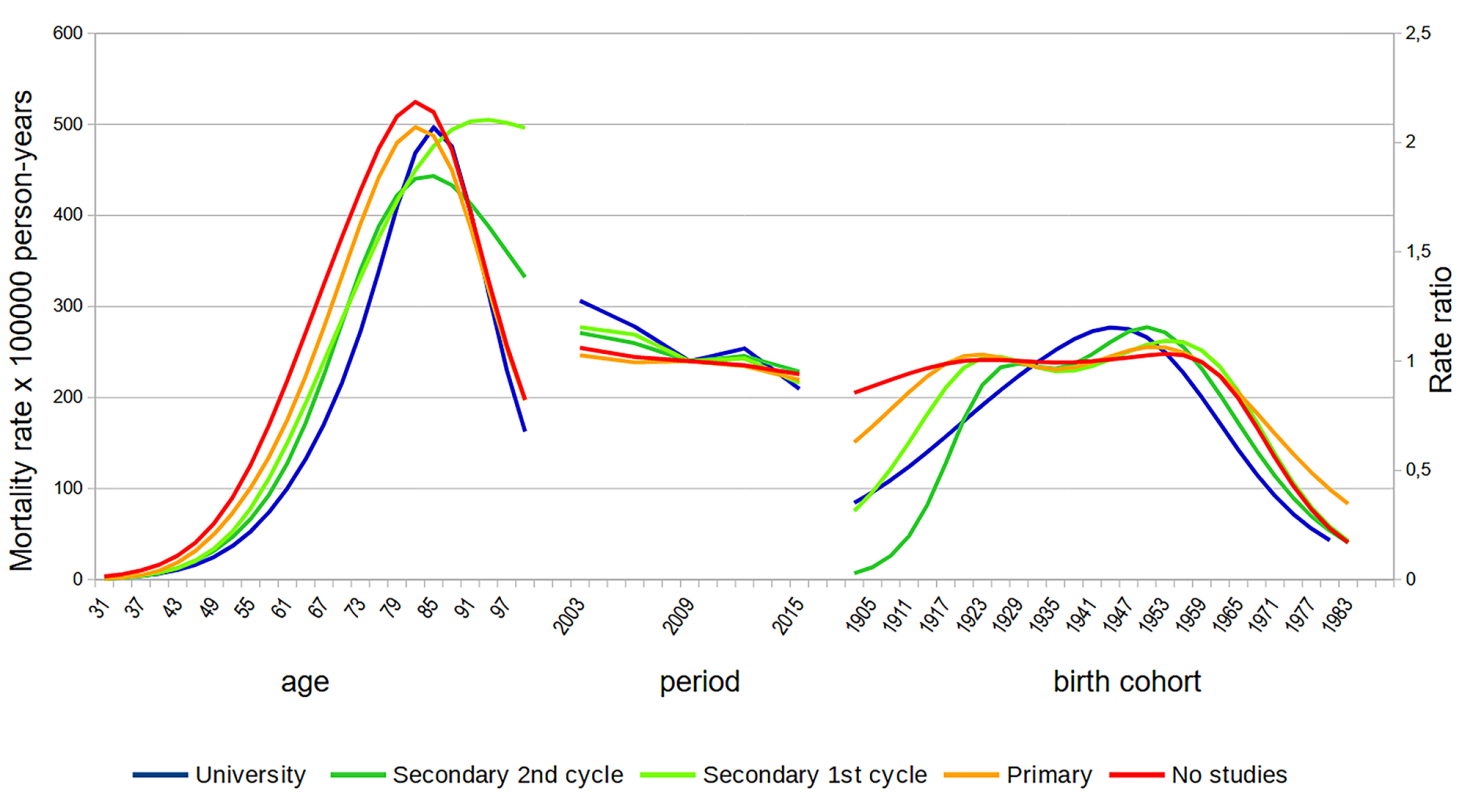
APC analysis of lung cancer mortality by educational level. Andalusian men, 2002–2016

**Fig. 7:**
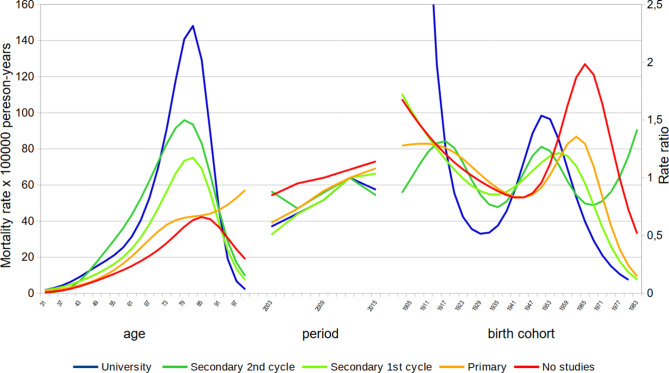
APC analysis of lung cancer mortality by educational level. Andalusian women, 2002–2016

We further analysed age, period and cohort effects by socioeconomic status, using educational level as the stratification variable. The rates were higher in men with lower educational attainment across all ages, with an almost perfect educational gradient up to age 80 (Fig. 6). The period effect decreased in all educational groups, but the slope of the decline was greater the higher the educational level, with a more dramatic decline in college-educated men. The cohort effect in men varied according to educational groups. In groups with lower educational attainment, the peak effect was reached in earlier cohorts. For example, those with no education reached it with the 1915 cohort; those with primary education, with the 1925 cohort; those with secondary education, with the 1932 cohort; and those with university education, with the 1940 cohort. In addition, the beginning of the decline of the effect followed a reverse order, with those with university studies beginning the decline with an earlier generation (1947), and those without education beginning the decline with a later birth cohort (1959).

In contrast, we observed a clear positive educational gradient in lung cancer death rates in women across all age groups (Fig. 7). The increase in rates with age was especially steep in the group of college-educated women between 60 and 80 years of age. An increase in the period effect was practically detected in all educational groups during the time covered by the study but, interestingly, a change in the trend was detected in the last period in the two groups of more educated women, in which the period effect started to decrease. Education groups showed important disparities in the cohort effect in women, but only from the 1940 birth cohort onwards. College-educated women peaked with the 1953 birth cohort, and began to decline with the younger cohorts. The lower the educational level, the later the cohort with which the decline began. This decline was not seen in women with no education until the 1968 birth cohort.

## Discussion

This paper highlights the different trends in lung cancer mortality by gender and educational level in Andalusia, a Southern European region. Our results show important differences by socioeconomic level that are expressed in age, period and cohort effects, and that need to be considered to disentangle their relationship with key causal factors, such as tobacco consumption and access to preventive and control interventions. Although there is considerable research on the association between economic status and lung cancer [8, 31], very little has been found in the literature on age-period-cohort analysis by socioeconomic status and lung cancer by gender.

The overall analysis by gender reveals much higher lung cancer mortality rates in men than in women over the whole period, although the sex ratio was halved from 14:1 in 2002–2004 to 7:1 in 2014–2016. This change is similar to that observed in other Southern European countries, where the evolution of the smoking pandemic is lagging behind in women compared to other Northern European countries or the United States of America [32, 33]. In the coming years, the difference between men and women will narrow, if the temporal pattern that we observed when comparing the period effect between both sexes in our study persists, with a clearly decreasing pattern in men and an increasing one for women.

The cohort effect also shows important differences between men and women. In fact, among men, the oldest cohorts experienced an increase in mortality, up to those born around 1920. From that cohort to those born in 1955, there was a plateau or stabilisation of the mortality rate, which declined steadily in subsequent cohorts. This pattern has been observed previously [34] and is related to the trend in smoking prevalence in men, which remained consistently high over a wide age range until 1985 [35]. In contrast, among women, a decline was seen in cohorts born up to 1935, which has been attributed to a reduction in environmental tobacco exposure among non-smoking women, progressively less exposed to tobacco smoke generated by their spouses at home [36]. Cohorts born after 1935 experienced an increase in mortality until 1960, related to the increase in smoking among women in Andalusia, which was practically negligible before 1970[35]. Moreover, we observed a decreasing lung mortality trend over birth cohort among women aged 30 to 47 years, probably as a result of an earlier decline in smoking prevalence among women in this age range, although the overall prevalence of smoking among Andalusian women did not start to decline until quite late, around 2002, well after the turning point in smoking prevalence among Andalusian men, circa 1985[34] .

Educational level was negatively associated with lung cancer mortality in all age groups in men, with the highest educated always experiencing the lowest rates. Despite this, it is men with a college education who showed the sharpest decline while those with no education show a stationary trend over the period of our research. This led to an increase in social inequalities in lung cancer mortality in men. This result is consistent with some recent literature that points to increasing inequalities by educational attainment and deprivation level [37]. Over the 15-year timespan of our study, the relationship between educational level and lung cancer mortality in women was positive, i.e. the rates were higher at higher educational levels. Furthermore, the analysis of the period effect shows an increase in mortality at all educational levels, with the exception of a change in the increasing trend in the two groups of more educated women in the last period. We could attribute the more pronounced downward trend in the more educated groups of men over the whole period, and of women in the last three years, to better utilisation or improved access to timely diagnosis and treatment in these subgroups or as a result of a greater global impact of public health recommendations on smoking cessation. As the fundamental cause theory holds, when knowledge of the causal relationship between smoking and lung cancer becomes more widespread and thus the disease increasingly preventable, those with greater access to resources such as power, knowledge or money, or who live in contexts of higher socio-economic status, will disproportionately benefit from the situation [37, 38].

Although the improvements in survival over the study period were not very striking (in Spain, survival in men was 11.2% in the 6-year period 2002–2007 and 12.7% in the 6-year period 2008–2013, while in women the change was from 16.2 to 17.6%) [39], it has been reported that patients with lower socioeconomic status also have poorer cancer survival, most likely due to delay in healthcare seeking [40] and lower likelihood of receiving traditional and next-generation treatments, in addition to higher comorbidity rates [41].

The most interesting finding to emerge from the analysis was the diverse cohort effect observed by educational level both in men and women. In men, the peak effect was reached earlier the lower the level of educational attainment, with a 25-year difference between the least educated and those with university educations. What is more striking is that the start in the decline of mortality followed the reverse pattern. Thus, university graduates men experienced declining rates with the birth cohort of 1947, i.e. 12 annual cohorts in advance of the least educated group, which started a downward trend in mortality rates with the 1959 cohort. We also observed educational differences in the cohort effects in women, with more rapid trend changes than men in all groups. Similarly to men, the lower the educational level, the later the cohort in which the downturn began. However, larger differences in the year of onset of decline were observed between men and women among the less educated groups (up to 8 or 9 cohorts apart), than among the more educated groups. As we previously stated, the cohort effect on lung cancer mortality refers to circumstances that can affect each entire generation, such as similar exposures to tobacco smoke, the main causal factor for lung cancer. Assuming similar latent periods in educational attainment and gender subgroups [42], the differences reported in the cohort effects in lung cancer mortality are mainly related to unequal exposures to tobacco in each cohort across the different socioeconomic and gender strata in men and women. Additionally, in older men with lower levels of education there is a clear effect of occupational exposures due to high past exposure to occupational carcinogens such as asbestos or heavy metals [43]. More specifically, in our study area, part of the results observed in men with low levels of education and advanced age (older cohorts) could be a consequence of past occupational exposures to carcinogens such as benzo(a)pyrene, asbestos, nickel, chromium and arsenic, established environmental risk factors for lung cancer [44], and with particularly relevant exposure burdens in the provinces of Cadiz, Huelva and Seville. These provinces have been highly industrialised for decades, and have been identified in several studies as high-risk areas for lung cancer, with a stable spatial pattern of mortality in men since the 1990s [45].

It can thus be suggested that there is excess mortality due to greater exposure to tobacco among men and women with a lower level of education, more so among women, who benefit from tobacco prevention and control measures with a significant delay. Indeed, numerous studies have pointed out that tobacco control measures have been gender [14, 46] and socially inequitable [15] – i.e. they have not been designed and implemented to reach and have an impact on the most vulnerable groups, which are those with the highest prevalence of tobacco consumption [47].

Indeed, we have observed a dissociation between the abundant evidence of the need for a gender and social determinants approach to the tobacco policies and the strategies implemented in many countries, including Spain [48]. As early as 2004, Mackenbach et al., after assessing inequalities in lung cancer mortality in several European countries, stated that it might not be too late to prevent the initiation of smoking among women of lower socio-economic levels in order to avoid greater inequalities in the future [13]. The results of our research point to the fact that the recommendations have not been followed. With very rare and new exceptions [49], even recent documents that inspire public policies on smoking still suffer from a lack of a gender-sensitive and social equitable approach [50, 51], in contrast to the tobacco companies’ successful strategies designed to bring women into smoking [14].

As was pointed out in the introduction to this paper, we were interested in the usefulness of choosing either an individual measure of socio-economic status or a measure of the contextual effects. From the results of the regression models, we found that the relative differences in lung cancer mortality using the contextual measure (deprivation index), although trending in the same direction, were clearly smaller in magnitude than those detected with the individual socioeconomic measure (education). This is still a matter of debate, because the concordance between ecological and individual measures is not well known [52]. Although there is evidence that contextual-level disparities play a role in cancer mortality [53], based on our results, we decided to restrict the assessment of inequality with the APC approach by using only the education variable as a measure of socio-economic status.

One limitation of this study is that we do not have information on lung cancer incidence data for the entire Andalusian population. There are several population-based provincial cancer registries that so far have not produced information disaggregated by socio-economic level [54].

Secondly, the limitations of using a census tract deprivation index should be highlighted. The most important limitation is the possibility of ecological fallacy, which could explain the differences with the results obtained using individual educational attainment. It is also possible that the deprivation index has a stronger association with lung cancer mortality in urban than in rural areas, an issue that has not been addressed in our study, which does not discriminate between rural and urban residence. In any case, aggregate deprivation indices are still considered quite good proxies for individual deprivation [55]. In our setting the deprivation index is also fairly stable over time, with minimal changes when classifying census tracts into quintiles over a period of a decade (data not shown). In addition, the LDAP assigns to each individual all changes in the census tract of residence during the period under study. The possibility of a gender bias could also be raised, as we are using a unique index for each census tract and no gender-sensitive variables are included in the construction of the index. In general, similar to ours, the most commonly used deprivation indices are indistinguishable for women and men residing in the same small area. Moreover, since the associations are in the same direction as those obtained for educational attainment for both sexes and in almost all periods, we are fairly confident in the robustness of our results.

The results of our APC analysis must been interpreted with caution. As we previously mentioned in the methods section, the main drawback of this analysis lies in the fact that the three variables involved are mathematically linked, the so-called identification or identifiability problem. Different approaches have been used to try to solve this limitation. Unfortunately, any procedure requires a great deal of theoretical knowledge and strong assumptions about one of the three effects, and none is able to completely overcome the identification problem [56]. Another limitation regarding APC analysis is that variables related to one of the components may also be related to another. For instance, advances in medical treatment for lung cancer might affect all ages, thus producing period effects, or have an influence on specific age groups that could extend over a lifetime, thus yielding cohort effects [56]. Finally, another source of weakness in our study regarding APC models is that some results must be interpreted with caution because recent cohort effects are usually calculated with small numbers and could lead to erroneous inferences.

## Conclusions

The present study has assessed socioeconomic inequalities in lung cancer mortality in a population cohort of 7.35 million individuals in a southern European country followed up between 2002 and 2016. Educational inequalities in lung cancer mortality in men increased due to an improvement in the more educated groups, among whom mortality was reduced in earlier cohorts, in relation to the decline in exposure to the main carcinogen, smoking. In women, there was an incipient reversal of the existing inequality in favour of those with less education, as it was among the most educated women that a reduction in mortality was observed in earlier cohorts. Our study reveals that APC analysis by education helps to uncover detailed changes in trends occurring in different socioeconomic and gender groups which, in combination with registers of smoking prevalence, provide important clues for action. Despite its limitations, this approach to the study of cancer inequalities allows for the assessment of gaps in historical and current tobacco policies and the identification of population groups that need to be prioritised for public health interventions.

## Data Availability

The data that support the findings of this study are available from Instituto de Estadística y Cartografía de Andalucía (IECA) but restrictions apply to the availability of these data, which were used under license for the current study, and so are not publicly available. Data are however available from the authors upon reasonable request and with permission of Instituto de Estadística y Cartografía de Andalucía.

## List of abbreviations

APC: Age-period-cohort
LDAP: Longitudinal Database of the Andalusian Population
